# First survey on the occurrence, subtype distribution and circulation of the enteric protozoan *Blastocystis* sp. in French broiler chicken farms

**DOI:** 10.1016/j.psj.2026.107332

**Published:** 2026-06-24

**Authors:** Constance Denoyelle, Laetitia Bonifait, Nausicaa Gantois, Ségolène Quesne, Louise Baugé, Jeremy Desramaut, Luen-Luen Li, Ruben Garcia Dominguez, Maëlle Lamisse, Magali Chabé, Franck Bonardi, Christophe Audebert, Sébastien Monchy, Gabriela Certad, Marianne Chemaly, Muriel Guyard-Nicodème, Eric Viscogliosi

**Affiliations:** a Univ. Lille, CNRS, Inserm, CHU Lille, Institut Pasteur de Lille, U1019 – UMR 9017 – CIIL – Centre d’Infection et d’Immunité de Lille, Lille, France; b ANSES, Laboratoire de Ploufragan Plouzané Niort, Site de Ploufragan, Unité UHQPAP, Ploufragan, France; c Université du Littoral Côte d’Opale, CNRS, Univ. Lille, UMR 8187, LOG, Laboratoire d’Océanologie et de Géosciences, Wimereux, France; d Centro Andaluz de Biología del Desarrollo, CSIC, Universidad Pablo de Olavide, Sevilla, Spain; e GD Biotech - Gènes Diffusion, Lille, France; f PEGASE-Biosciences, Institut Pasteur de Lille, Lille, France; g Délégation à la Recherche Clinique et à l’Innovation, Groupement des Hôpitaux de l’Institut Catholique de Lille, Lille, France

**Keywords:** *Blastocystis* sp., Intestinal protozoa, Broiler chicken, Molecular epidemiology, Circulation

## Abstract

*Blastocystis* sp. is the most commonly detected enteric protozoan in humans, yet its presence in broiler chickens remains insufficiently characterized despite its clear zoonotic potential. Here, we present the first survey conducted in France—and the largest to date in Europe—aimed at assessing the frequency, subtype (**ST**) and genotype distribution, and circulation patterns of *Blastocystis* sp. in broiler chicken production. A total of 967 cecal samples were collected at a single slaughterhouse from 66 farms representing three production systems (Label Rouge, standard, and certified). *Blastocystis* sp. was detected by real-time PCR in 49.7% of samples and in 83.3% of farms. Statistical analyses identified production system—reflecting differences in bird age and outdoor access—together with season, as major factors associated with parasite prevalence. Among mono-colonizations characterized by Sanger sequencing, ST7 was by far the most predominant ST, followed by ST6, reinforcing their avian origin and their capacity to co-circulate within farms. Genotyping revealed substantial genetic diversity within ST7 and moderate diversity within ST6. The dominance of specific ST7 and ST6 genotypes, potentially “adapted” to broiler chickens, suggests extensive chicken-to-chicken transmission, whereas rarer genotypes could reflect sporadic contamination from transient wild bird species that frequently harbor the parasite. Mixed colonizations involving at least two STs were detected in nearly 20% of positive animals. Next-generation sequencing of selected samples confirmed either co-colonization with ST6 and ST7 or the presence of highly divergent ST7 genotypes. Although avian STs are generally considered non-pathogenic in poultry, this is not the case in humans. Consequently, the active circulation of *Blastocystis* sp. in French broiler chickens highlights the potential significant zoonotic reservoir represented by these flocks. Transmission could occur through direct contact between poultry workers and birds, through ingestion of water contaminated with bird feces, or potentially via handling of contaminated carcasses—an understudied route that warrants further investigation.

## Introduction

Recent large-scale molecular epidemiology studies consistently show that *Blastocystis* sp. is a ubiquitous protozoan, colonizing the gastrointestinal tract of hundreds of millions of people worldwide, making it the most frequently encountered unicellular eukaryote in the human population (Andersen and Stensvold, 2016). It is also found in a wide diversity of animal hosts, ranging from insects to mammals, including birds, mollusks, fish and reptiles among others (Yoshikawa et al., 2016; Gantois et al., 2020; Rauff-Adedotun et al., 2020; Hublin et al., 2021, 2021; Ryckman et al., 2024). Primarily transmitted via the fecal–oral route (Tan, 2008), notably through the consumption of water or food contaminated with fecal material containing cystic forms of the parasite, *Blastocystis* sp. can reach prevalence rates exceeding 50% in populations from developing countries, in association with poor sanitary conditions and inadequate water quality (Khaled et al., 2020; Guilavogui et al., 2022).

One of the main characteristics of this protozoan is its marked genetic diversity, as revealed by comparisons of small subunit ribosomal DNA (**SSU rDNA**) gene sequences. To date, 45 lineages, referred to as subtypes (**STs**), have been validated, each of which may potentially correspond to a distinct species (Figueiredo et al., 2025). To our knowledge, 18 of these 45 STs have been to date identified in humans, with highly variable frequencies (Alfellani et al., 2013; Jinatham et al., 2021; Osorio-Pulgarin et al., 2021; Hernandez-Castro et al., 2023; Naguib et al., 2023; Aung et al., 2025). Consistent with a predominantly human to human transmission, ST1 to ST4 account for more than 90% of subtyped human isolates (Alfellani et al., 2013; Popruk et al., 2021; Rauff-Adedotun et al., 2021; Jimenez et al., 2023), with an overall predominance of ST3 and pronounced geographic variations in the prevalence of other STs, particularly ST4. The remaining STs identified in humans are considered to be of animal origin, suggesting zoonotic transmission. While most of these animal-adapted STs remain minor in the human population, this is not the case for ST6 and ST7, which are classically regarded as avian STs due to their high prevalence in wild and domestic birds (Hublin et al., 2021; Rauff-Adedotun et al., 2021; Mohammadi et al., 2025; Pavlickova et al., 2025; Rehena et al., 2025). Accordingly, ST7 is by far the most frequent zoonotic ST in the human population, highlighting the significant impact of animal-to-human transmission (Stensvold and Clark, 2016). This mode of transmission has been clearly demonstrated, for example, by the identification of identical genetic variants of ST6 in broiler chickens and in workers from the corresponding slaughterhouse (Greige et al., 2018).

The impact of zoonotic transmission is also clinically relevant, as the pathogenicity of this protozoan—most commonly manifested by diarrhea and abdominal pain—appears to be largely ST-dependent. This association has been consistently reported in both experimental models (*in vitro* and *in vivo*) (Ajjampur and Tan, 2016; Deng and Tan, 2025) and human cohort studies (Deng et al., 2022).Thus, the virulence of avian ST7 isolates has been demonstrated by their ability to adhere to and disrupt the gut epithelial barrier, resulting in increased intestinal permeability. Under the same experimental conditions, these deleterious effects on the intestinal epithelium were not observed for ST4 isolates, which are predominantly associated with human carriage. Furthermore, the zoonotic ST7 has been implicated in the induction of gut dysbiosis through an increase in pathogenic bacterial populations and promotion of immune dysregulation. In contrast, colonization by ST1 to ST4—predominant in the human population—has generally been associated with beneficial effects, including greater gut microbiota diversity, increased abundance of beneficial bacterial taxa and enhanced anti-inflammatory activity (Deng et al., 2021, 2022, 2023; Antonetti et al., 2024; Huang et al., 2024).

To date, epidemiological investigations into the prevalence of *Blastocystis* sp. in avian hosts remain scarce, particularly in chickens (Hublin et al., 2021; Rauff-Adedotun et al., 2021; Mohammadi et al., 2025; Pavlíčková et al., 2025; Rehena et al., 2025). This knowledge gap is notable given the major role of the poultry sector in France, which provides approximately 100,000 direct and indirect jobs. Furthermore, the French broiler chicken population—distributed across more than 8,500 farms—currently numbers around 145 million animals (Agreste, 2025). Broiler chicken consumption has also risen steadily over recent years, reaching an average annual per capita intake of approximately 25 kg. In parallel, chickens constitute a potential high-risk reservoir for zoonotic transmission due for example, to their frequent close contact with farm and abattoir workers and the widespread use of poultry feces as fertilizer on agricultural land used for human food production. Consequently, exposure to live infected animals or poultry carcasses could represent an occupational hazard for chicken industry workers and a potential risk to consumers.

Consequently, the striking lack of epidemiological data on the impact of *Blastocystis* sp. in poultry prompted us to conduct the first study in France, as well as the largest survey ever carried out in Europe, on the frequency and distribution of *Blastocystis* sp. STs and genotypes in broiler chicken farms. The study also evaluated the influence of several factors—including production system (and associated animal age and outdoor access) and seasonality —on the circulation of this protozoan, while assessing the potential role of chickens as reservoirs of infection for humans.

## Materials and methods

### Ethics statement

All broiler chicken samples were collected after slaughter at the evisceration stage by slaughterhouse staff. No approval from the Institutional Animal Care and Use Committee or ethics committee was necessary, as no experiments involving live animals were performed.

### Study sites and sample collection

This large-scale survey was conducted over one year (2024–2025) in France to evaluate potential seasonal effects on the prevalence of *Blastocystis* sp. in broiler chickens all belonging to the subspecies Gallus gallus domesticus. Samples were collected at a single slaughterhouse and originated from 66 different broiler farms. (Table S1). In France, broiler production is divided into several quality segments, including standard, certified, and Label Rouge systems (Beaumont et al., 2004). Standard production is an intensive system using fast-growing strains reared indoors at high stocking densities and slaughtered at around 35–42 days of age, without outdoor access. Certified production represents an intermediate system with stricter specifications, including a minimum slaughter age of approximately 56 days, lower stocking densities, and enhanced feed and traceability requirements. However, birds are generally reared indoors, with no mandatory outdoor access and, when present, outdoor areas are typically very limited in size. In contrast, the Label Rouge system relies on slow-growing strains, mandatory outdoor access from around 6 weeks of age, lower stocking densities, and a minimum slaughter age of 81 days. The study encompassed these three distinct poultry production systems: standard (31 farms), certified (7 farms), and Label Rouge (28 farms). For each farm shipment upon arrival at the slaughterhouse, additional information was obtained from the mandatory ICA/FCI (Food Chain Information) form completed prior to slaughter, notably regarding animal health status. In this survey, all chickens appeared clinically healthy upon arrival at the slaughterhouse, with no evidence of diarrhea at the time of sampling. Flocks arriving at the slaughterhouse comprised approximately 4,200 to 48,500 chickens, depending on the farm. A total of 84 chicken batches were collected. For 70 batches, 10 chickens were sampled; for 13 batches, 20 chickens; and for one batch, 30 chickens, amounting to a total of 990 chickens. Over the study period, 52 of the 66 farms were sampled once, 11 farms twice, 2 farms three times, and 1 farm four times. Briefly, the entire intestinal tract was collected from randomly selected animals in each batch at the evisceration area by slaughterhouse personnel. Upon arrival at the laboratory, only the ceca of each animal were aseptically removed, and their contents were transferred to a sterile plastic container. To prevent sample degradation prior to DNA extraction, an equal volume of 2.5% potassium dichromate solution (w/v in water) (Sigma Life Sciences, Saint Louis, MO, USA) was added to cecal contents prior to storage at 4°C. For farms sampled more than once, the detection of a single *Blastocystis* sp.–positive sample in one of the batches was sufficient to classify the farm as positive. Samples with insufficient quantity of cecal content (n = 23) were excluded from further analysis. In total, 967 samples corresponding to the three production systems and collected over the four seasons were validated and analyzed.

### DNA extraction and detection of *Blastocystis* sp. by real-time PCR

The first step involved removing the potassium dichromate from the cecal contents by performing three washes with distilled water following centrifugation at 3,000×g for 10 min. DNA was then extracted from 500 µL of the washed cecal contents resuspended in 1 mL of distilled water using a commercial kit (NucleoSpin 96 Soil, Macherey-Nagel GmbH & Co KG, Düren, Germany) according to the manufacturer’s protocol and subsequently stored at 4 °C until use. The identification of the protozoan in these samples was performed by real-time PCR (**qPCR**) using 2 µl of extracted DNA and the primer pair BL18SPPF1 (5′-AGT AGT CAT ACG CTC GTC TCA AA-3′) / BL18SR2PP (5′-TCT TCG TTA CCC GTT ACT GC-3′), allowing the amplification of an approximately 300 bp fragment of the SSU rDNA gene of all known *Blastocystis* sp. STs (Poirier et al., 2011). Cecal samples were tested in duplicate, with positive (*Blastocystis* sp. ST4 strain WR1 DNA) and negative (nuclease-free water) controls included in each qPCR run. Samples were considered negative if the cycle threshold (**Ct**) exceeded 36 or no amplification occurred. Ct values were recorded for all positive samples, and the corresponding amplicons were purified and sequenced using Sanger technology (Genoscreen, Lille, France). For a substantial number of cecal samples, overlapping sequence chromatograms revealed possible mixed colonizations with the presence of at least two distinct *Blastocystis* sp. STs in the same animal, which could not be resolved by Sanger sequencing. SSU rDNA gene sequences from samples exhibiting single ST carriage were deposited in GenBank under accession numbers PX634771–PX635159. These sequences were compared against all publicly available homologous sequences corresponding to known *Blastocystis* sp. STs in the National Center for Biotechnology Information (NCBI) database using the Nucleotide Basic Local Alignment Search (BLASTN) tool (https://blast.ncbi.nlm.nih.gov/Blast.cgi). STs were assigned based on exact or closest matches. BioEdit version 7.7.1 (https://bioedit.software.informer.com/) (Hall, 1999) was used to align sequences within each ST, assess intra-ST diversity, and define representative genotypes for phylogenetic analyses.

### Phylogenetic inference

Phylogenetic analyses were conducted to corroborate the ST assignments obtained by BLASTN for the *Blastocystis* sp. isolates analyzed in this study. The full-length SSU rDNA gene reference sequences of the 45 STs validated to date (Figueiredo et al., 2025) were first retrieved from GenBank and supplemented with 6, 20, and 4 additional full-length sequences available for ST6, ST7, and ST9, respectively, to enhance phylogenetic resolution within these clades and to facilitate accurate placement of the newly characterized isolates. This panel of homologous sequences was aligned using the program MAFFT version 7.490 (https://mafft.cbrc.jp/alignment/software/) using the iterative refinement L-INS-i method (Katoh and Standley, 2013). A maximum-likelihood (**ML**) phylogenetic tree was subsequently inferred from this alignment using IQ-TREE (http://www.iqtree.org/) under the best-fitting nucleotide substitution model (K2P+I+G4), with branch support assessed by 1,000 bootstrap replicates (Nguyen et al., 2015). The tree was rooted on the ST15–ST28 cluster reported as the most basal lineage within the *Blastocystis* genus (Santin et al., 2024; Figueiredo et al., 2025). The 24 partial SSU rDNA gene sequences representing all genotypes identified from single colonizations in the present survey were subsequently included to the same MAFFT reference alignment then placed within the fixed ML tree using the highly scalable tool EPA-ng (Barbera et al., 2019). The resulting data were analyzed with Gappa (Czech et al., 2020) and the online tool Interactive Tree of Life iTOL version 7 (https://itol.embl.de/) was ultimately used for the visualization and annotation of the tree (Letunic and Bork, 2021).

### Sequence identity matrix and network-based analysis of genotypes

Pairwise nucleotide identity analyses were performed for *Blastocystis* sp. ST6 and ST7 to evaluate sequence similarity among the genotypes identified in this study and reference sequences. Identity estimates were based on the same SSU rDNA gene multiple-sequence alignment used for phylogenetic inference. The alignment was restricted to the region corresponding to the amplified fragment, defined as the span between the first and last alignment positions containing at least one unambiguous nucleotide (A, C, G or T) among the sample sequences. ST-specific alignments for ST6 and ST7 were extracted and further curated by removing alignment columns composed exclusively of gap characters across all sequences, while retaining columns containing gaps in at least one sequence. Pairwise nucleotide identity was calculated as the proportion of identical nucleotide positions between two sequences, considering only sites where both sequences contained unambiguous nucleotides, following a pairwise deletion approach for gap and ambiguous positions. Identity values were expressed as proportions ranging from 0 to 1 and visualized as heat maps. An initial alignment including only the genotypes identified in this study for ST6 and ST7 was subsequently analyzed in R version 4.1.0 (https://www.R-project.org/) (R Core Team, 2023) to infer and visualize haplotype / genotype networks using the geneHapR package version 1.2.4 (Zhang et al., 2023). Sample-associated metadata were integrated to investigate the distribution of haplotypes / genotypes across season and production type.

### Next-generation amplicon sequencing

To identify the STs in reported cases of *Blastocystis* sp. mixed colonizations detected by Sanger sequencing, ten cecal samples were randomly selected from two farms, and the corresponding SSU rDNA amplicons were sequenced using the minION platform (Oxford Nanopore Technologies, Oxford, UK). Briefly, libraries were prepared with the Native Barcoding Kit (Oxford Nanopore Technologies) following the manufacturer’s instructions. Amplicons were end-repaired and dA-tailed using the NEBNext Ultra II End Repair/dA-Tailing Module (NEB, Ipswich, MA, USA), barcoded with the Blunt/TA Ligase Master Mix (NEB), and ligated to sequencing adapters using the NEBNext Quick Ligation Module (NEB). Purified libraries were loaded onto a MinION flow cell and sequenced. After demultiplexing and adapter removal, reads were taxonomically screened using BLASTN against the SILVA 18S rDNA database (version 138; https://www.arb-silva.de/). Reads identified as *Blastocystis* sp. were subsequently assigned to specific STs using BLASTN against a custom reference database comprising representative sequences from all 45 currently recognized STs included in the phylogenetic analysis, applying a stringent E-value cutoff of 1e-100. STs representing less than 1% of total reads per sample were excluded from the analysis. Next-generation sequencing (**NGS**) data have been deposited in the NCBI Sequence Read Archive (SRA) under BioProject accession number PRJNA1425029.

### Statistical analysis

All statistical analyses were performed using R version 4.5.2 (R Core Team, 2023). Associations between categorical variables were assessed using Pearson’s chi-squared (**χ²**) tests. When appropriate, Fisher’s exact test was used for pairwise comparisons between categories. The variables investigated included production type, season, *Blastocystis* sp. status, STs and genotypes. To identify factors associated with *Blastocystis* sp. presence, a logistic generalized linear mixed model (GLMM) was fitted using the lme4 package version 1.1-37. Covariates included season and production type, while farm was included as a random effect. Model outputs were reported as odds ratios (**OR**) with 95% confidence intervals (**CI**) using the finalfit package version 1.0.8 (https://finalfit.org/). A threshold of *P* ≤ 0.05 was considered statistically significant.

## Results and discussion

### Occurrence of *Blastocystis* sp. in French broiler chicken and assessment of parasitic load

To fill the existing gap in epidemiological data regarding *Blastocystis* sp. frequency in broiler chicken, the first survey conducted in France—and the largest in Europe—was performed by screening a total of 967 cecal samples from animals originating from 66 French broiler farms (Table S1). The sample size in this study was similar to that of the Chinese largest global survey conducted in chickens to date, which screened 1,000 birds from 43 farms (Liu et al., 2022). Of the 967 French broiler chicken samples screened by qPCR for the presence of *Blastocystis* sp., 481 tested positive, corresponding to a substantial prevalence of 49.7% (Table 1). Accordingly, this prevalence substantially exceeded the average colonization rate of approximately 24% previously reported in chickens (Rehena et al., 2025). Considering only the few surveys conducted in broiler chickens that included a substantial number of animals (over 100) and used molecular diagnostics for *Blastocystis* sp. (Table 1), the occurrence observed in France is the highest reported worldwide, markedly surpassing frequencies of 14.5% in Turkey (Onder et al., 2021), 17.0% in Egypt (Naguib et al., 2022), 20.1% (Liu et al., 2022) and 26.3% (Feng et al., 2024) in China, 26.0% in Malaysia (Noradilah et al., 2017), 31.8% in Lebanon (Greige et al., 2018), and 33.8% in Brazil (Maloney et al., 2021).

**Table 1:** Frequency and distribution of *Blastocystis* sp. STs in the largest chicken cohorts analyzed by molecular methods worldwide.

Country	No. of samples	No. of positive samples (percentage prevalence)	No. of farms (if available)	No. of positive farms (percentage prevalence)	*Blastocystis* sp. STs (No. of isolates)	References
Turkey	220	32 (14.5)			ST7 (32)	Onder et al., 2021
Egypt	217	37 (17.0)			ST7 (36) ST14 (1)	Naguib et al., 2022
Lebanon	223	71 (31.8)	74	48 (64.9)	ST6 (55) ST7 (16)	Greige et al., 2018
Brazil^a^	130	44 (33.8)			ST6 (23) ST7 (43) ST10 (1) ST14 (5) ST25 (1) ST29 (2)	Maloney et al., 2021
China	1000	201 (20.1)	43	30 (69.8)	ST6 (44) ST7 (157)	Liu et al., 2022
China	617	162 (26.3)	18	16 (88.9)	ST6 (20) ST7 (137) ST10 (3) ST23 (2)	Feng et al., 2024
Malaysia	104	27 (26.0)			ST1 (3) ST3 (7) ST6 (2) ST7 (12) ST9 (3)	Noradilah et al., 2017
France	967	481 (49.7)	66	55 (83.3)	ST6 (92) ST7 (295) ST4 (2) MI^b^ (92)	Present study

a Number of positive samples lower than the number of subtyped isolates due to the analysis of mixed infections using next-generation amplicon sequencing.

b Mixed infection.

The analyzed French broiler chickens originated from 84 batches, of which 66 (78.6%) were positive for *Blastocystis* sp. Among these positive batches, 42 exhibited animal colonization rates exceeding 70%, and 31 showed 100% of positivity among the analyzed samples, although the sampled chickens were randomly selected upon arrival of the flocks at the slaughterhouse. Moreover, of the 11 farms sampled twice at different time points, both batches were positive in four farms, whereas both were negative in one farm; in the remaining six farms, one batch was positive and the other negative. Among the two farms sampled three times, two batches were positive and one was negative. For the single farm sampled four times, all batches tested positive. When batch-level results were aggregated at the farm level, 55 of the 66 French farms (83.3%) were positive for *Blastocystis* sp. For comparison, based on the limited number of surveys reporting the farm origin of analyzed broiler chicken, similarly high proportions of infected farms have been reported, reaching 69.8% (30/43) (Liu et al., 2022) and 88.9% (16/18) (Feng et al., 2024) in China, and 64.9% (48/74) (Greige et al., 2018) in Lebanon (Table 1). Taken together, these data clearly indicate active circulation of *Blastocystis* sp. in broiler chicken farms and extensive colonization of animals by this protozoan particularly in France.

Such high colonization levels in French broiler chickens prompt consideration of parasite burden, as elevated intestinal loads may drive substantial fecal shedding, thereby enhancing intra-flock transmission and increasing the risk of environmental contamination in poultry farms. Colonization intensity was roughly estimated from Ct values obtained for each qPCR-positive broiler chicken sample. Although no quantification curve specific to the STs identified in the present survey was available, most positive samples showed low Ct values, including a minimum Ct value of 5 (Table S1) and a mean Ct value of 17 ± 6. As an initial assessment, extrapolation from the available ST3 calibration data (Sejnohova et al., 2024) suggests that most chicken samples harbored high intestinal parasite burdens, with 79.0% exhibiting Ct values below 20 and 58.6% falling within the Ct range of 5–16. By contrast, Ct values measured in colonized bivalves using the same qPCR assay as that employed in the present study have never been reported below 20 (Ryckman et al., 2026). This pattern is consistent with the role of mollusks as passive carriers of *Blastocystis* sp., reflecting environmental accumulation rather than multiplication of the protozoan within the host. In comparison, the low Ct values observed in French broiler chickens are suggestive of high intestinal colonization levels associated with active multiplication of the protozoan. However, this interpretation remains tentative and should be confirmed through additional investigations, particularly histological examination of the gastrointestinal tract of infected birds.

### Parameters influencing *Blastocystis* sp. incidence in French broiler chickens

The first factor evaluated was the broiler chicken production system, with sampling comprising 474 standard, 155 certified, and 338 Label Rouge animals (Table 2). Based on our global data, the prevalence of *Blastocystis* sp. differed substantially among production systems (**χ²** test, *P* ≤ 0.0001). Indeed, the risk of protozoan colonization was significantly higher in Label Rouge (302/338, 89.3%) than in certified (105/155, 67.7%; Fisher’s exact test, *P* ≤ 0.0001) and standard (74/474, 15.6%; Fisher’s exact test, *P* ≤ 0.0001) broiler chickens (Table 2). Likewise, certified chickens were significantly more frequently colonized than standard poultry (Fisher’s exact test, *P* ≤ 0.0001).

**Table 2:** Frequency and distribution of *Blastocystis* sp. STs reported according to the production system of broiler chickens.

			*Blastocystis* sp. STs No. of isolates (%)			
Production system	No. of samples/No. of positive	Prevalence (%)	ST6	ST7	ST4	MI^a^
Label Rouge	338/302	89.3	45 (14.9)	207 (68.5)	0 (0)	50 (16.5)
Certified	155/105	67.7	26 (24.8)	56 (53.3)	0 (0)	23 (21.9)
Standard	474/74	15.6	21 (28.4)	32 (43.2)	2 (2.7)	19 (25.7)
Total	967/481	49.7	92 (19.1)	295 (61.4)	2 (0.4)	92 (19.1)

a Mixed infection.

To our knowledge, this is the first survey to assess the impact of different chicken production systems on *Blastocystis* sp. colonization and to determine their contribution to parasite prevalence in farms. The elevated parasite prevalence in Label Rouge chickens, along with the gradual increase across production systems, likely reflects multiple contributing factors. Foremost is the longer rearing period, which extends stepwise from the standard to the certified and ultimately to the Label Rouge systems. Accordingly, increasing age thus appears to be a major factor promoting *Blastocystis* sp. colonization in French chicken production units, as well as in Chinese free-range broiler farms (Liu et al., 2022). Conversely, another survey conducted in China reported a higher prevalence of *Blastocystis* sp. in younger free-range chickens compared with older animals (Feng et al., 2024). Broadly speaking, the longer an animal lives, the greater the likelihood of exposure to parasites present in its environment. Moreover, according to reports in the literature, age is also an important factor in cattle and pigs, with a significantly higher prevalence of this protozoan in older animals (Hublin et al., 2021; Rehena et al., 2025).

Another factor that could account for the higher prevalence of *Blastocystis* sp. in Label Rouge chickens is their access to outdoor environments—an option unavailable in standard production systems and potentially extremely restricted in certified farms. Indeed, outdoor access represents a potential source of soil and water contamination through the droppings of wild birds, which are themselves frequently colonized by the parasite (Hublin et al., 2021; Rauff-Adedotun et al., 2021; Mohammadi et al., 2025; Pavlíčková et al., 2025; Rehena et al., 2025). Additional sources of exposure include cysts shed in feces by chickens from the same cohort or by other farm animals and subsequently ingested through coprophagous behavior, as well as potentially infected insects that are consumed as part of the poultry diet (Yoshikawa et al., 2016). In contrast, animal crowding in standard systems does not appear to be a key driver of transmission since the prevalence of *Blastocystis* sp. is inversely correlated with animal density on farms.

Seasonality, in direct interaction with production systems and access to outdoor areas, may also play a non-negligible role in the circulation of *Blastocystis* sp. within broiler chicken farms. Based on our sampling (262 samples collected in spring, 243 in summer, 307 in autumn, and 155 in winter), a clear seasonal effect was observed in the overall animal population (χ² test, *P* ≤ 0.0001) (Table 3), with the prevalence of *Blastocystis* sp. in spring (154/262, 58.7%), and summer (140/243, 57.6%), and, to a lesser extent, autumn (142/307, 46.3%) being significantly higher than that observed in winter (45/155, 29.0%) (Table 3). This effect was particularly pronounced when comparing the warm summer period with the cold winter season across all production systems (Fisher’s exact test, *P* ≤ 0.0001), as follows: Label Rouge chickens (94.4% in summer versus 75% in winter), certified chickens (60% in summer versus 45% in winter), and standard chickens (25.9% in summer versus 6.3% in winter). A similar seasonal trend has also been reported in a survey of broiler chickens in Lebanon (Greige et al., 2018). For Label Rouge chickens, and to a much lesser extent for certified animals, the warm and humid conditions characteristic of spring and summer, combined with outdoor access, may influence exposure to *Blastocystis* sp. by favoring the survival and persistence of cysts in the environment, particularly in soil and water sources, thereby increasing the risk of transmission (Moe et al., 1996). These favorable seasons are also associated with a higher abundance of insects, which can act as mechanical vectors, and with increased activity and density of wild birds near poultry farms, potentially contributing to environmental contamination through fecal shedding. For standard chickens reared entirely indoors, this seasonal pattern was evident, with a marked decline in prevalence during winter. During hot periods exceeding the thermal comfort zone for broilers (18–24 °C) and inducing heat stress, birds markedly reduce feed intake and increase water consumption as part of their physiological thermoregulatory response. This behavior contributes to higher litter moisture due to more liquid excretions and water spillage, as well as increased ambient humidity through panting and respiratory evaporation (May and Lott, 1992; Lawal et al., 2024). These conditions likely favor, at least indirectly, the environmental persistence and transmission of enteric parasites such as *Blastocystis* sp. Conversely, colder winter conditions, together with better control of litter moisture and improved on-farm hygiene practices, are likely to reduce the environmental survival of parasite cysts, thereby lowering infection pressure, particularly in standard chickens.

**Table 3:** Seasonal effect on the frequency of *Blastocystis* sp. in French broiler chickens production systems.

		
Season/Production system	No. of samples/No. of positive	Prevalence (%)
**Total winter**	**155/45**	**29.0**
Label Rouge	40/30	75.0
Certified	20/9	45.0
Standard	95/6	6.3
**Total spring**	**262/154**	**58.7**
Label Rouge	118/100	84.7
Certified	70/49	70.0
Standard	74/5	6.8
**Total summer**	**243/140**	**57.6**
Label Rouge	90/85	94.4
Certified	45/27	60.0
Standard	108/28	25.9
**Total autumn**	**307/142**	**46.3**
Label Rouge	90/87	96.7
Certified	20/20	100
Standard	197/35	17.8

Collectively, these data, analyzed using a multivariate approach, highlight a multifactorial influence on the prevalence of *Blastocystis* sp. in broiler chicken populations. Production system characteristics (certified: OR = 148.94, CI: 9.75–2275.63, *P* ≤ 0.001; Label Rouge: OR = 1086.39, CI: 131.28–8990.11, *P* ≤ 0.001), particularly poultry age and outdoor access, together with seasonal effects—especially the difference between spring and winter (OR = 0.05, CI: 0.01–0.23, p ≤ 0.001)—played a major combined role in parasite circulation.

### Distribution of *Blastocystis* sp. STs from mono-colonizations in broiler chickens

**Fig. 1:**
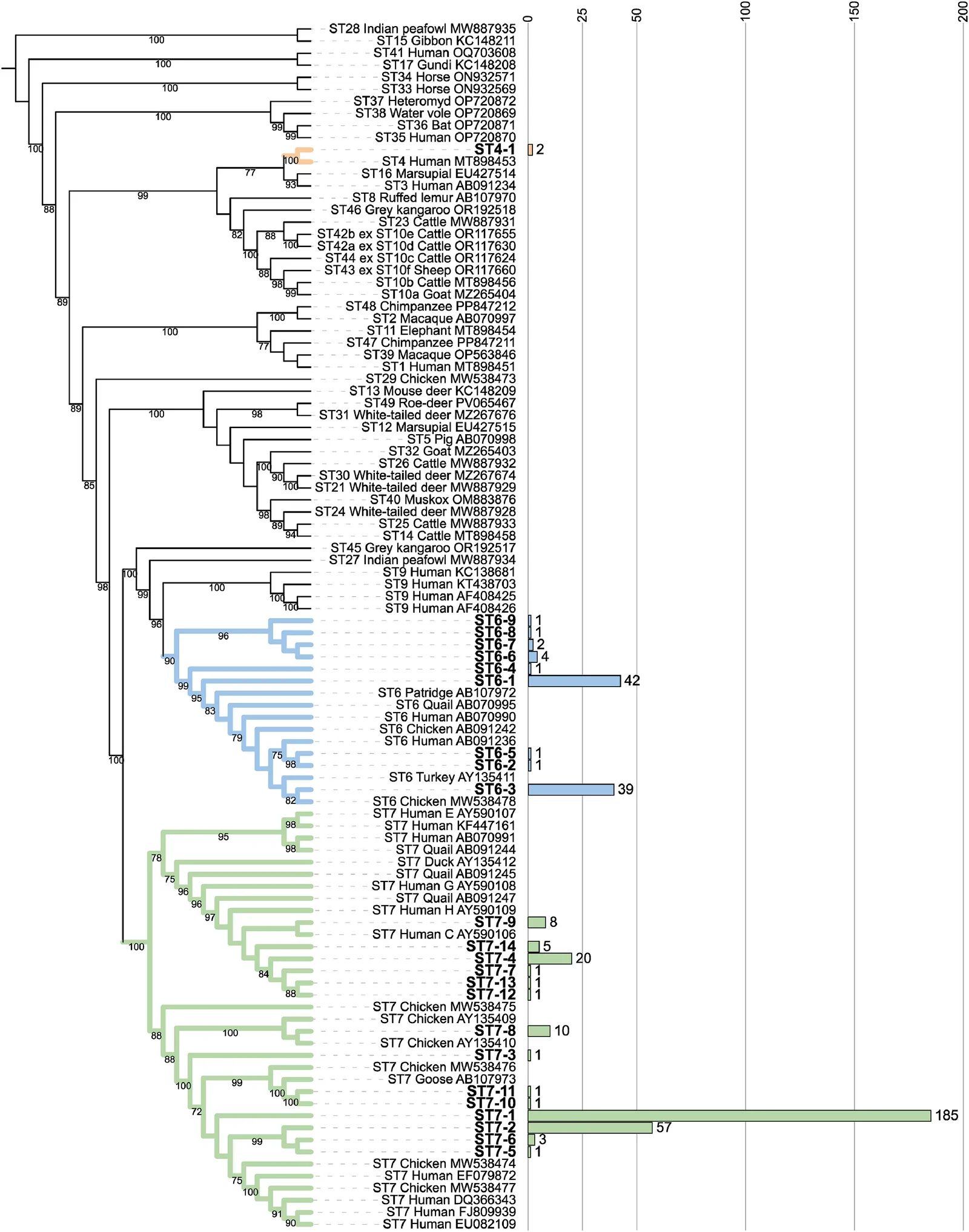
Maximum-likelihood phylogenetic reconstruction of *Blastocystis* sp. isolates inferred from partial SSU rDNA gene sequences. Accession numbers of reference sequences representing the 45 currently accepted STs are provided. Genotypes identified in this study from French broiler chicken samples are highlighted in bold**.** The number of isolates per genotype within ST6, ST7, and ST4 is indicated by blue, green, and red boxes, respectively. Bootstrap support values are displayed at the nodes and only values ≥70% are shown.

Analysis of Sanger sequencing chromatograms from the 481 chicken cecal samples that tested positive for *Blastocystis* sp. by qPCR revealed that 389 (80.9%) corresponded to single-ST carriage. BLASTN database searches showed that the sequences obtained shared 97.1-100% identity with available homologous sequences of the protozoan in the GenBank database. Furthermore, alignment of these 389 sequences identified a total of 24 genetic variants or genotypes, each comprising a highly variable number of chicken isolates (Fig. S1). Preliminary BLASTN-based ST assignment indicated that 14 genotypes belonged to ST7, 9 to ST6, and 1 to ST4. To confirm this initial classification, all 24 genotypes were included in a comprehensive phylogenetic analysis incorporating the reference sequences of the 45 known *Blastocystis* sp. STs (Fig. 1). The topology of the resulting ML tree, rooted on the most basal ST15–ST28 cluster, was overall similar to that reported in the most recent study based on comparisons of full-length SSU rDNA gene sequences (Figueiredo et al., 2025). The phylogenetic analysis strongly confirmed the initial BLASTN-based identification of the chicken isolates, as the proposed ST7, ST6, and ST4 genotypes clustered with their corresponding reference sequences with bootstrap support values of 100%, 90%, and 100%, respectively.

ST7 therefore exhibited a large predominance in the French broiler chicken cohort, with 295 isolates identified, followed by ST6 (92 isolates) and ST4 (2 isolates) (Table 1 and Fig. 1). When considering only mono-colonizations, the relative frequencies of these STs among the subtyped isolates were 75.8% (295/389) for ST7, 23.7% for ST6 (92/389), and 0.5% for ST4 (2/389). Consistent with previous studies, chickens—and birds more broadly—serve as natural hosts of ST6 and ST7 which clearly represent avian STs, supporting the notion of host specificity for these STs (Hublin et al., 2021; Mohammadi et al., 2025; Pavlickova et al., 2025; Rehena et al., 2025). Interestingly, the co-occurrence of ST7 and ST6, accompanied by a predominance of ST7, has been reported in large-scale molecular epidemiological surveys conducted for instance in Brazil (Maloney et al., 2021), China (Liu et al., 2022; Feng et al., 2024), and Malaysia (Noradilah et al., 2017) (Table 1), as well as in smaller-scale studies involving a limited number of chickens, such as those carried out in the Philippines (Adao and Rivera, 2024) and Thailand (Jinatham et al., 2021; McCain et al., 2023). ST7 was also the only avian ST identified in several surveys, including those conducted in Egypt (Naguib et al., 2022), Turkey (Onder et al., 2021), Côte d’Ivoire (D’Alfonso et al., 2017), and Italy (Gabrielli et al., 2021). As indicated in Table 1, Lebanon is unique among all studied countries in exhibiting a predominance of ST6, which accounts for 77.5% of isolates compared with 22.5% for ST7 (Greige et al., 2018). This pattern suggests that geographic factors may shape the distribution of these STs in chickens, even though ST7 is otherwise largely dominant worldwide.

Within the framework of our study, the ratio of the number of ST7 isolates to ST6 isolates identified in mono-colonizations was significantly highly dependent on the production system (χ² test, *P* ≤ 0.001) (Table 2). Indeed, it was of 4.6 (207/45) for Label Rouge chickens, 2.1 (56/26) for certified animals, and 1.5 (32/21) for standard chickens (Fisher’s exact tests: Label Rouge versus Certified *P* ≤ 0.05, Label Rouge versus Standard *P* ≤ 0.01 and Certified versus Standard *P* ≤ 0.44). Correlated with variations in parasite occurrence according to the production system detailed above, these differences in the ST7/ST6 ratio could be directly linked to the potential access of animals to the outdoors, representing a possible source of contamination via wild bird droppings, which are predominantly colonized by ST7 (Hublin et al., 2021; Mohammadi et al., 2025; Rehena et al., 2025). Nevertheless, the co-circulation of ST6 and ST7 was detected in 11 of the 66 batches of chickens positive for *Blastocystis* sp.***,*** corresponding to 10 farms. This proportion is very likely substantially underestimated due both to the limited number of chickens analyzed per farm and to the lack of full consideration of mixed colonizations.

In addition to ST6 and ST7, other STs have occasionally been identified in chickens in various countries. These include ST1, ST3, and ST9 in Malaysia (Noradilah et al., 2017), ST2 in Australia (Roberts et al., 2013), ST5 in Brazil (Valença-Barbosa et al., 2019), ST10 in Brazil, China, and Iran (Rostami et al., 2020; Maloney et al., 2021; Feng et al., 2024), ST14 in Egypt, Brazil, and Iran (Rostami et al., 2020; Maloney et al., 2021; Naguib et al., 2022), ST23 in China (Feng et al., 2024), ST25 in Brazil (Maloney et al., 2021) and finally ST29 in Brazil and Thailand (Maloney et al., 2021; Tantrawatpan et al., 2023). With the exception of ST9 and ST29, which may be poultry-adapted (Tantrawatpan et al., 2023; Pavlíčková et al., 2025), the other STs are not considered as host-specific of chickens and are therefore most likely the result of cross-species transmission. Indeed, although ST1–ST3 have been identified in various animal groups (Hublin et al., 2021), they are primarily found in humans (Popruk et al., 2021; Rauff-Adedotun et al., 2021). ST5 is mainly associated with pigs and ST10, ST14, ST23, and ST25 are predominantly reported in herbivores (Pavlíčková et al., 2025). In the present study, two ST4 isolates were identified from standard chickens raised on two different farms. To our knowledge, this is the first report of the presence of ST4 in chicken worldwide. As previously stated, ST4 is frequently detected in humans (Popruk et al., 2021; Rauff-Adedotun et al., 2021), and therefore transmission of the protozoan would be plausible through the contact between chickens and human fecal material. Notably, the sequences of these two ST4 isolates showed 100% identity with those of human isolates reported in approximately fifteen countries, including France (isolate ET35, Accession No. KU158949) (El Safadi et al., 2016). However, this hypothesis appears unlikely given the sanitary conditions and staff protection measures required in French standard poultry farms. Because of the overall predominance of ST4 in rodents (Hublin et al., 2021), a second hypothesis would be that frequent and well-documented rodent incursions into farm buildings may result in poultry contamination through contact with rodent feces. Indeed, poultry farm environments, regardless of the production system, provide favorable conditions for rodent proliferation by offering nesting sites and access to food. Supporting this second hypothesis, sequences from rodent isolates, including rats (strain WR1, Accession no. AY590113; isolate S1, Accession no. AY590111) (Noël et al., 2005), exhibit 100% identity with the two ST4 isolates identified in this study.

### Diversity, distribution and circulation of *Blastocystis* sp. ST6 and ST7 genotypes in the French chicken farms

The 92 aligned partial SSU rDNA gene sequences obtained from ST6 isolates in the present study ranged from 277 to 278 bp in length and revealed 18 variable positions, defining nine distinct genotypes (ST6-1 to ST6-9) as stated above (Fig. S1). Regarding the 295 ST7 isolate sequences, their lengths ranged from 296 to 302 bp, with the identification of 42 variable sites representing 14 genotypes (Fig. S1). Pairwise identity matrices calculated between sequences of these genotypes and those of reference isolates belonging to ST6 or ST7, and visualized as heat maps (Fig. S2), revealed a very high level of intra-ST genetic diversity, particularly for ST7. Indeed, based on pairwise comparisons of partial SSU rDNA gene sequences, ST7 sequences exhibited identity values ranging from 91.2% to 100%. These identity percentages were slightly higher for ST6 isolates, ranging from 94.9% to 100%. A sequence divergence cut-off of 4% between STs was initially proposed (Stensvold and Clark, 2020) even if this threshold was recently lowered to 2–3% in the subdivision of ST10 into three new STs (Santin et al., 2024). Although this issue of *Blastocystis* sp. isolate classification lies outside the scope of the present study, our data clearly highlight the need to subdivide these two avian STs into additional STs in order to differentiate sequence variants within these clades. As our qPCR assay targets only an approximately 300-bp region of the SSU rDNA gene, such ST6 and ST7 subdivisions cannot yet be proposed. To confirm the validity of these potential ST divisions, near-complete SSU rDNA gene sequences from the relevant ST6 and ST7 isolates should be generated and compared with homologous full-length sequences for both STs.

**Fig. 2:**
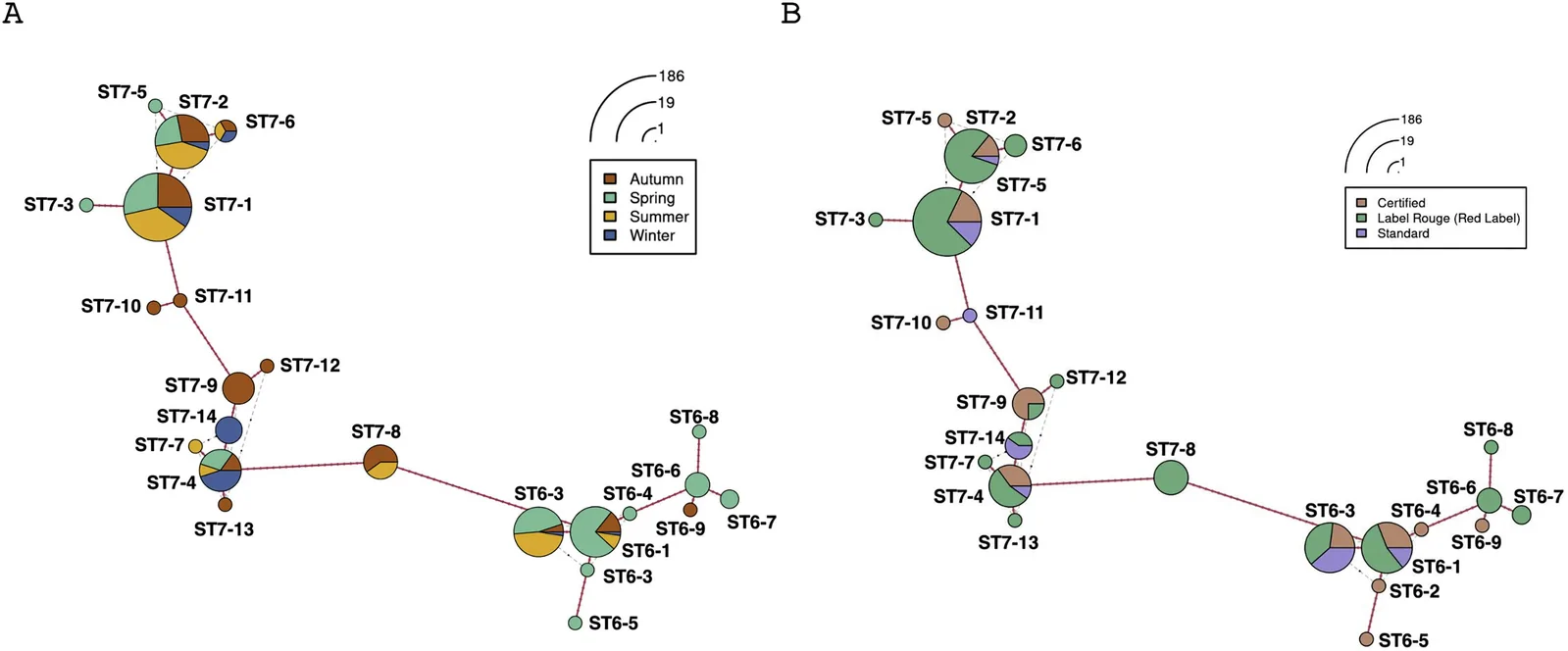
Network analysis of *Blastocystis* sp. ST6 and ST7 genotypes identified in French broiler chickens according to production system (A) and season (B). The size of each pie chart is proportional to the total number of isolates corresponding to each genotype. Colors within the pie charts represent the proportion of isolates for each genotype according to the parameters considered, as indicated in the legend. The length of the links between genotypes is correlated with the number of mutational steps separating the genetic variants.

Among the 14 ST7 genotypes (Fig. 1), ST7-1 accounted for nearly 63% (185/295) of the isolates and was identified in 36 out of the 55 farms positive for *Blastocystis* sp*.* A second major, though less prevalent, genotype ST7-2 included 19.3% of the isolates (57/295) and was detected in 21 farms. For ST6, two genotypes, ST6-1 and ST6-3, together accounted for nearly 90% of isolates (81/92), occurring cumulatively across 20 farms. The remaining genotypes of both STs were much less frequent, with 12 genotypes represented by a single isolate each. Among the 52 farms positive for *Blastocystis* sp. that did not exclusively present mixed colonizations (Table S1), a single ST6 or ST7 genotype was identified in 16 farms; two genotypes in 22 farms; three genotypes in seven farms; four genotypes in five farms; five genotypes in one farm; and nine genotypes in a single farm. These genotype frequency data, combined with network analyses (Fig. 2) aimed at determining the distribution of genotypes according to production system and season and subsequently, to formulate hypotheses regarding the circulation patterns of *Blastocystis* sp. within these French poultry farms. Analysis of the genotype networks first confirmed the greater genetic diversity of ST7 compared with ST6 (Fig. 2), consistent with previous molecular analyses of *Blastocystis* sp.–positive isolates from chickens in Brazil (Maloney et al., 2021) and China (Liu et al., 2022). This was reflected in the structure of the networks, with a longer cumulative link between genotypes as well as a higher number of genotype clusters potentially representative of new STs (4 for ST7: ST7-1, −2, −3, −5, −6; ST7-10, −11; ST7-4, −7, −9, −12, −13, −14 and ST7-8 versus 2 for ST6: ST6-1, −2, −3, −4, −5 and ST6-6, −7, −8, −9). In addition, the poultry production system appeared to be a major determinant of genetic diversity in terms of genotype richness (χ² test, *P* ≤ 0.001), with a total of 16 genotypes (11 ST7 and 5 ST6) identified in Label Rouge chickens, compared with only 12 (6 ST7 and 6 ST6) in certified chickens and 7 (5 ST7 and 2 ST6) in standard chickens (Fig. 2A). This observation strongly supports the hypothesis proposed above that outdoor access facilitates exposure to a broader diversity of environmental sources, particularly through fecal contamination by resident or migratory wild birds carrying diverse *Blastocystis* sp. genotypes. In this context, Passeriformes, which represent more than half of all avian species, are primarily infected with ST6, whereas ST7 appears to be the dominant ST in Anseriformes (Hublin et al., 2021). These genotypes, introduced sporadically into poultry farms, may subsequently persist at low frequency within chicken populations. The observed seasonal variation also reinforces this statement, as genotypic diversity peaked in spring and autumn (13 ST6 and ST7 genotypes per season), two seasons corresponding to the migration periods of wild birds, but declined markedly in winter (7 genotypes), a period during which outdoor access is largely restricted. Strikingly, the four major genotypes identified in our study (ST7-1, ST7-2, ST6-1, and ST6-3), in terms of the number of isolates, were the only ones detected in chickens, regardless of production system or season (Fig. 2). Moreover, in 31 of the 52 *Blastocystis* sp.–positive farms without exclusively mixed colonizations (Table S1), chickens were colonized solely by one or more of the four major genotypes. Isolates belonging to these same genotypes are also the only ones that have previously been reported in chicken farms in Lebanon (Greige et al., 2018) and, in the case of ST7-1, also in Egypt (Naguib et al., 2022) and Turkey (Onder et al., 2021). Furthermore, isolates of ST6-1, which predominate in French poultry farms, have also been reported as predominant in Lebanese farms (Greige et al., 2018). Collectively, these findings strongly suggest that, within avian hosts, the four major genotypes identified in the present study could be specifically “adapted” to broiler chickens and actively circulate within French poultry farms, and potentially worldwide. Conversely, the remaining ST6 and ST7 genotypes detected on these farms could reflect incidental contamination from feces of other transient avian species colonized by *Blastocystis* sp., and found at low frequencies within chicken populations. This hypothesis should be validated through large-scale epidemiological studies in broiler chicken farms across a broad range of countries.

### Mixed colonizations and next-generation amplicon sequencing

Analysis of Sanger sequencing chromatograms revealed a substantial frequency of mixed colonizations among French broiler chicken samples. Specifically, 91 of 481 samples (18.9%) potentially harbored at least two *Blastocystis* sp. STs. Notably, none of the major epidemiological studies that subtyped isolates using this sequencing approach reported mixed-ST carriage (Table 1). The frequency observed in our survey may, at least in part, be explained by the high prevalence of the parasite and the broad genetic diversity of the subtyped isolates, many of which likely originate from external avian contamination, thereby increasing opportunities for mixed colonizations. To obtain a near-complete overview of the *Blastocystis* sp. STs colonizing chickens in France, NGS was applied to ten cecal samples randomly selected from two farms and exhibiting mixed colonizations. SSU rDNA amplicons were sequenced using Nanopore technology, generating between 300,000 and 1,200,000 reads per sample after quality filtering and first screening, for a total of over 8.5 million reads analyzed (Table S2). BLASTN analysis against a custom reference database comprising all currently known *Blastocystis* STs identified two samples from the same farm exhibiting co-carriage by ST6 and ST7, with a marked predominance of ST7-associated reads. This co-carriage was expected, given the near-exclusive presence of these two avian-associated STs in French broiler chicken farms. By contrast, more than 99.5% of the reads obtained from the remaining eight samples were assigned exclusively to ST7, an observation that initially appeared inconsistent with the presence of mixed colonizations. This apparent inconsistency likely reflected the limited representation of ST7 diversity in the reference database, which included only a single ST7 sequence despite substantial genetic variability among ST7 isolates. Consequently, the co-occurrence of distinct ST7 genotypes within the same isolate—potentially corresponding, as suggested above, to novel STs— could therefore account for sequencing chromatogram patterns resembling co-carriages. In this regard, in the second farm in which four mixed colonizations were analyzed, three ST7 genotypes (ST7-2, ST7-6, and ST7-14), forming two well-separated evolutionary clusters in our phylogenetic tree (Fig. 1), were reported in single-ST carriages. Despite the sequencing depth achieved with this method, no additional STs were identified in chickens, thereby confirming the strong predominance and near exclusivity of ST6 and ST7 in France. In Brazil, a similar sequencing approach revealed four additional STs alongside ST6 and ST7 among free range chickens (Maloney et al., 2021), three of which are thought to originate from herbivores. These differences between the two countries seems to be correlated with farming practices and the potential contact with livestock as reported for chickens in Brazil.

### Implications of *Blastocystis* sp. occurrence for poultry health and zoonotic transmission

Even though identifying gastrointestinal symptoms in poultry farms remains complex—except perhaps for diarrhea—all epidemiological studies conducted to date and focusing on *Blastocystis* sp. are focused on cohorts of healthy chickens (Table 1). Overall, in avian species, only a single case of *Blastocystis* sp. pathogenicity has been reported to date in the literature in which ST7 was found in a duck causing subcutaneous rather than enteric infection (Ilchyshyn and Monti, 2018). Taken together, these findings, combined with the high prevalence of *Blastocystis* sp. observed in our study and the likely high parasite burden in colonized animals, point to intense circulation within poultry farms without evidence of pathogenicity. Despite its intestinal tropism and the frequency and intensity of colonization, the potential impact of *Blastocystis* sp. on the bacterial gut microbiota of broiler chickens remains unclear as data are currently lacking. Nevertheless, two recent studies conducted in chickens in Lebanon (Greige et al., 2019) and Egypt (Guyard-Nicodème et al., 2023) have shown that this parasite may influence gut microbiota composition, with *Blastocystis* sp. infection being associated with increased colonization by Campylobacter spp. Ongoing studies aim to clarify the role of *Blastocystis* sp. as a modulator of the bacterial gut microbiota in chickens colonized by ST7 or ST6 and to elucidate the mechanisms underlying its association with distinct bacterial communities.

The high prevalence of *Blastocystis* sp. in French broiler chickens also raises the question of the risk of zoonotic transmission of avian ST6 and ST7. Such transmission has already been confirmed, for example by the characterization of identical ST6 isolates in a slaughterhouse among broiler chickens and staff, despite the implementation of regulatory protective measures in these facilities (Greige et al., 2018). Moreover, isolates corresponding to three of the four predominant genotypes identified in our survey (ST7-1, ST6-1, and ST6-3) have already been reported in human populations in numerous countries. Focusing on Europe, isolates assigned to these three genotypes have been identified in the Czech Republic (Lhotská et al., 2020) and Poland (Kaczmarek et al., 2017; Rudzińska and Sikorska, 2023), while isolates corresponding to ST7-1 and ST6-1 have been described in Cyprus (Seyer et al., 2017), ST6-1 in Serbia (Süli et al., 2021) and Italy (Mattiucci et al., 2016), and, interestingly, ST6-3 in France (El Safadi et al., 2016). In addition, isolates of less prevalent genotypes in French chickens have already been reported in humans, such as ST7-8 in the Czech Republic and Poland (Kaczmarek et al., 2020; Lhotská et al., 2020), and ST7-14 in the Czech Republic (Accession No. MT042799). From the cumulative epidemiological data presented by Stensvold and Clark (2016), both avian STs are estimated to represent approximately 10% of human *Blastocystis* sp. colonizations. Moreover, cases of diarrhea have been reported in Serbian patients colonized by the ST6-1 genotype (Süli et al., 2021), as well as in both a dog and its owner, who were infected with the ST7-8 genotype (Kaczmarek et al., 2020). More recently, ST7 was identified exclusively in diarrheic children in China (Zhao et al., 2024). Taken together, these data strongly suggest that chickens, and more broadly birds, could represent a significant source of zoonotic transmission of *Blastocystis* sp. and constitute a major potential risk to human health. However, the impact of human infection with avian STs is very likely underestimated, as *Blastocystis* sp. remains too often overlooked or not routinely investigated in stool samples from patients presenting with digestive disorders.

In our opinion, the observed frequency of avian STs in the human population cannot be explained solely by a single factor, such as frequent contact between chickens and farmers or slaughterhouse workers. Moreover, very few studies have focused on the level of transmission risk to professionals in the poultry sector, despite their close relationship with these animals or their constant exposure to feces. Other sources of transmission should therefore be investigated, such as the consumption of water contaminated by bird droppings and carcass handling by poultry industry professionals or, more commonly, by consumers. To date, no investigations have assessed the presence of *Blastocystis* sp. on eviscerated carcasses intended for consumption while improperly performed evisceration of chickens could lead to fecal contamination, potentially posing a risk to handlers lacking adequate protective equipment. More globally, it is imperative to raise awareness among broiler chicken farmers about environmental sources of contamination in order to limit the spread of *Blastocystis* sp., and to promote parasite monitoring in poultry farms so as to better inform and protect poultry industry professionals who receive only limited medical follow-up and, ultimately, the consumer population.

## Author Contribution

Ségolène Quesne and Louise Baugé coordinated the sampling schedule at the slaughterhouse, collected all relevant information associated with the samples, collected caecal samples, prepared them for storage, and managed their shipment to the Lille team. Constance Denoyelle, Nausicaa Gantois, Jérémy Desramaut, and Maëlle Lamisse performed DNA extraction from all biological samples, conducted real-time PCR assays, and analyzed the resulting PCR data. Constance Denoyelle and Ruben Garcia Dominguez performed sequence alignments and phylogenetic analyses. Frank Bonardi and Christophe Audebert conducted network-based analyses of genotypes and statistical analyses. Luen-Luen Li and Sébastien Monchy performed next-generation amplicon sequencing and subtype assignment of sequencing reads. Laetitia Bonifait, Magali Chabé, Gabriela Certad, Marianne Chemaly, Muriel Guyard-Nicodème, and Eric Viscogliosi conceived and designed the study and/or supervised the overall experimental work. Marianne Chemaly, Muriel Guyard-Nicodème, Laetitia Bonifait, and Eric Viscogliosi secured funding for the study. Constance Denoyelle and Eric Viscogliosi drafted the original manuscript, while Marianne Chemaly and Muriel Guyard-Nicodème coordinated and completed the relevant submission procedures. All authors critically reviewed the manuscript, provided feedback, and approved the final version.

## Disclosures

The authors declare that they have no known competing financial interests or personal relationships that could have appeared to influence the work reported in this paper.

## Appendix Supplementary materials

Image, application 1

Image, application 2

Image, application 3

## Footnotes

Supplementary material associated with this article can be found, in the online version, at doi:10.1016/j.psj.2026.107332.
