# Supplementary material for: First survey on the occurrence, subtype distribution and circulation of the enteric protozoan Blastocystis sp. in French broiler chicken farms
Source: Poult Sci. 2026 Jun 24;105(11):107332. doi: 10.1016/j.psj.2026.107332 (PMC13572524; doi:10.1016/j.psj.2026.107332)
Supplement: Supplementary file 1 [file mmc3.pdf]

Table S2. Assignment of reads generated by next-generation sequencing from 10 broiler chicken samples identified as mixed infections by Sanger sequencing.

Sample number

	**751**		**752**		**754**		**756**		**759**		**760**		**455**		**456**		**457**		**458**	
**ST**	**No. of reads**	**%**	**No. of reads**	**%**	**No. of reads**	**%**	**No. of reads**	**%**	**No. of reads**	**%**	**No. of reads**	**%**	**No. of reads**	**%**	**No. of reads**	**%**	**No. of reads**	**%**	**No. of reads**	**%**
ST1	11	0,001	10	0,001	20	0,001	11	0,001	17	0,001	16	0,002	8	0,001	6	0,001	12	0,002	6	0,002
ST2	9	0,001	6	0,001	7	0,001	4	0,000	9	0,001	11	0,001	5	0,001	5	0,001	11	0,002	3	0,001
ST3	9	0,001	6	0,001	7	0,001	6	0,001	4	0,000	4	0,000	2	0,000	5	0,001	7	0,001	1	0,000
ST4	53	0,005	57	0,008	101	0,008	60	0,005	86	0,007	80	0,008	49	0,009	32	0,008	60	0,009	33	0,011
ST6	1662	0,142	151719	20,506	101213	7,589	2274	0,193	4042	0,340	4267	0,421	47	0,008	24	0,006	42	0,007	33	0,011
ST7	1167465	99,849	587686	79,432	1232243	92,396	1177187	99,799	1185090	99,648	1009661	99,567	568693	99,978	417932	99,981	645464	99,978	306641	99,975
ST9	7	0,001	359	0,049	51	0,004	9	0,001	10	0,001	7	0,001	-	-	-	-	1	0,000	-	-
ST21	2	0,000	3	0,000	1	0,000	1	0,000	1	0,000	-	-	1	0,000	-	-	-	-	-	-
ST27	-	-	-	-	-	-	-	-	1	0,000	1	0,000	-	-	-	-	2	0,000	-	-
ST26	-	-	-	-	-	-	-	-	-	-	1	0,000	1	0,000	-	-	-	-	-	-
ST30	9	0,001	10	0,001	5	0,000	5	0,000	11	0,001	6	0,001	10	0,002	6	0,001	5	0,001	2	0,001
ST45	-	-	6	0,001	3	0,000	-	-	2	0,000	2	0,000	3	0,001	2	0,000	1	0,000	-	-
Total	1169227	100,000	739862	100,000	1333651	100,000	1179557	100,000	1189273	100,000	1014056	100,000	568819	100,000	418012	100,000	645605	100,000	306719	100,000
