# Supplementary material for: First survey on the occurrence, subtype distribution and circulation of the enteric protozoan Blastocystis sp. in French broiler chicken farms
Source: Poult Sci. 2026 Jun 24;105(11):107332. doi: 10.1016/j.psj.2026.107332 (PMC13572524; doi:10.1016/j.psj.2026.107332)
Supplement: Supplementary file 2 [file mmc1.docx]

**Fig. S1.** Alignment of partial SSU rDNA gene sequences from *Blastocystis* sp. ST6 (A) and ST7 (B) isolates showing only variable sites. Variable positions relative to the arbitrarily selected reference sequence (genotypes ST6-1 and ST7-1) are indicated above the alignment (vertical numbering). Dashes denote nucleotides identical to the reference sequences, and asterisks indicate gaps. ST6 and ST7 genotypes are listed on the left, while the number and percentage of isolates identified for each genotype in this survey are shown on the right. For each identified ST6 and ST7 genotype, the percentage identity to the closest homologous Blastocystis sp. isolate sequence in GenBank, as determined by BLASTN with 100% query coverage, is reported in the rightmost column of the table.

```
A

     1111111111222222
    123345579999011246
	    782320813489647280

ST6-1	    TAATTTGTAGCGT*GAGA  42/92 (45.6%		100%
ST6-2	    ......A......*....   1/92 (1.1%)		99.6%
ST6-3	    ....C........*....  39/92 (42.4%)		100%
ST6-4	    ...........T.*....   1/92 (1.1%)		99.6%
ST6-5	    ......A......*.CTC   1/92 (1.1%)		98.6%
ST6-6	    ..TA.C.AGTAT.C....   4/92 (4.3%)		99.6%
ST6-7	    G*TA.C.AGTAT.C....   2/92 (2.2%)		99.3%
ST6-8	    ..TA.CAAGTATACTCT.   1/92 (1.1%)		97.8%
ST6-9	    ..TC.C.AGTAT.C....   1/92 (1.1%)		97.1%


B

            11111111111111111111111222222222222222
        389934445557777888888889999111111123333488
        290170162361234234567890135234567861456148

ST7-1   TTTGTGTTGTAACATTTTTATAAAAGTTTGACTTTTTGTGAT  185/295 (62.7%)	100%
ST7-2   ..................................G.......   57/295 (19.3%)	100%
ST7-3   ........T.................A............T.A    1/295 (0.3%)	98.7%
ST7-4   ...C..ACT..G...AAA.TGT.**T..*.....GG***...   20/295 (6.8%)	99.7%
ST7-5   ..................................G....C..    1/295 (0.3%)	99.7%
ST7-6   ..................................G.....G.    3/295 (1.0%)	99.7%
ST7-7   ...C..ACT..G...AAA.TG..**T..*.....GG***...    1/295 (0.3%)	99.7%
ST7-8   GAGTGCG.TGGGTG.GCA.T.GT**..G*TTAGG.G***...   10/295 (3.4%)	100%
ST7-9   ...C..GCT..G...AAA.TGTG**T..*......G***...    8/295 (2.7%)	99.7%
ST7-10  ............GTA...*..G......C......G**A.G.    1/295 (0.3%)	99.0%
ST7-11  ............GTA...*..G......C......G**A...    1/295 (0.3%)	99.3%
ST7-12  ...C..GCT..G...AAA.TGTG**T..*.....GG***.G.    1/295 (0.3%)	99.0%
ST7-13  ...C..GCT..G...AAA.TGT.**T..*.....GG***...    1/295 (0.3%)	99.7%
ST7-14  ...C..ACT..G...AAA.TGT.**T..*......G***...    5/295 (1.7%)	100%
```
